# Nature and severity of dental malocclusion in children suffering from transfusion-dependent (-thalassemia major

**DOI:** 10.1590/2177-6709.25.6.26.e1-9.onl

**Published:** 2020

**Authors:** Waqar Jeelani, Uroosa Sher, Maheen Ahmed

**Affiliations:** 1 Bakhtawar Amin Medical and Dental College, College of Dentistry (Multan, Pakistan).; 2 Sundas Foundation, Blood Bank and Hematological Services Charitable Organization (Sialkot, Pakistan).

**Keywords:** Thalassemia, Beta-thalassemia, Index of orthodontic treatment need, Malocclusion

## Abstract

**Objective:**

To evaluate the prevalence and severity of malocclusion in children suffering from β-thalassemia and to assess orthodontic treatment need using Grainger’s Treatment Priority Index (TPI) and index of orthodontic treatment need (IOTN)-dental health component (DHC).

**Methods:**

A cross-sectional study was conducted on 200 transfusion-dependent children diagnosed with homozygous β-thalassemia and 200 healthy school children aged 11-17 years. The TPI and IOTN-DHC data was recorded for both groups. Total TPI score for each subject was calculated and graded according to malocclusion severity estimate (MSE). Independent sample *t*-test was used to compare mean TPI scores, overjet and overbite between thalassemic and healthy children. Chi-square test was used to compare the frequency of IOTN-DHC grades, Angle’s classification, and MSE grades between thalassemic and healthy children.

**Results:**

The most prevalent malocclusion was Class I in normal children (67.5%) and Class II in thalassemic children (59%). The mean overjet and overbite were significantly (*p*<0.001) greater in thalassemic children than in healthy children. Severe tooth displacements were 3.5 times greater in thalassemic children, compared to controls. A greater proportion of thalassemic children were in IOTN grades 3 and 4, compared to the controls (*p*<0.001). MSE grades 4 and 5 were significantly (*p*<0.001) more prevalent in thalassemic children, compared to the controls.

**Conclusion:**

There is a high prevalence of Angle’s Class II malocclusion in thalassemic children. Majority of these children are categorized in higher grades of IOTN-DHC and TPI-MSE, showing a great severity of malocclusion and high orthodontic treatment needs.

## RESUMO

**Objetivo:**

Avaliar a prevalência e gravidade da má oclusão em crianças que sofrem de beta-talassemia e mensurar a necessidade de tratamento ortodôntico usando o Índice de Prioridade de Tratamento (IPT) de Grainger e o Índice de Necessidade de Tratamento Ortodôntico (*Index of Orthodontic Treatment Need* - IOTN) - Componente de Saúde Dental (*Dental Health Component* - DHC).

**Métodos:**

Um estudo transversal foi conduzido com 200 crianças com diagnóstico de beta-talassemia homozigótica dependentes de transfusão e 200 crianças saudáveis em idade escolar, entre 11 e 17 anos. Os dados do IPT e do IOTN-DHC foram documentados para ambos os grupos. A pontuação total do IPT para cada sujeito foi calculada e classificada de acordo com a Estimativa de Severidade da Má oclusão (ESM). O teste *t* para amostras independentes foi usado para comparar os escores médios de IPT, sobressaliência e sobremordida, entre as crianças saudáveis e as com talassemia. O teste qui-quadrado foi usado para comparar a frequência dos escores do IOTN-DHC, a classificação de Angle e os escores do ESM entre crianças com beta-talassemia e crianças saudáveis.

**Resultados:**

A má oclusão mais prevalente foi a Classe I em crianças normais (67,5%) e a Classe II em crianças com beta-talassemia (59%). Os valores médios de sobressaliência e a sobremordida foram significativamente (*p*< 0,001) maiores em crianças com beta-talassemia do que em crianças saudáveis. Os deslocamentos dentários graves foram 3,5 vezes maiores em crianças com beta-talassemia em comparação com os controles. Uma proporção maior de crianças com beta-talassemia estava nos escores 3 e 4 do IOTN em comparação com os controles (*p* <0,001). Os escores 4 e 5 de ESM foram significativamente (*p*< 0,001) mais prevalentes em crianças com beta-talassemia em comparação com os controles.

**Conclusão:**

Há uma alta prevalência de má oclusão de Classe II de Angle em crianças com beta-talassemia. A maioria dessas crianças é categorizada em escores superiores de IOTN-DHC e IPT-ESM, mostrando uma grande gravidade de má oclusão e alta necessidade de tratamento ortodôntico.

## INTRODUCTION

Thalassemia is one of the commonest types of hemoglobinopathies resulting from decreased synthesis of different types of polypeptide chains constituting normal adult hemoglobin molecule (HbA, α2 β2).^1^ β -thalassemia is one of the severest forms of this disease, resulting from mutations in the HBB gene on chromosome 11, which ultimately leads to partial or completely nonfunctional formation of β-globin chain. Depending on the severity and type of genetic mutations, β-thalassemias may be divided into β-thalassemia major, intermedia and minor.^2^

With a global prevalence of about 1.5%, the *per annum* incidence of β-thalassemia has been recorded to be 1 in 100,000 worldwide.^3^ Children suffering from β-thalassemia major comprise the most affected group and usually need regular blood transfusions. According to one estimate, about 5,000-9,000 transfusion-dependent β-thalassemia children are born every year in Pakistan.^4^ According to World Health Organization (WHO), Pakistan has the largest numbers of thalassemia major children in the world, a majority of which do not have access to the proper health facilities.^5^

In a β-thalassemia afflicted child, the diagnostic signs and symptoms start to become eminent within the first two years of life. The common clinical manifestations of β-thalassemia major usually originate from a severe form of chronic anemia associated with cardiac complications and hepatic pathologies.^6^ ^,^ ^7^ A striking abdominal enlargement by the virtue of enlarged spleen and liver is frequently observed.^7^ Moreover, extramedullary hematopoiesis and rapid cell turnover with consequential bone marrow expansion is a prominent feature. As a complication of iron overload, stunted growth as well as skeletal deformities with resultant osteoporosis and pathologic fractures are common.^8^

Regarding the pathognomonic orofacial features, the predominant ones include characteristic “Cooley’s facies”, marked by prominent frontal and parietal bones, saddle nose, protruded zygomas and epicanthic fold.^9^ The common dental features include Class II malocclusion, spacing, flaring and protrusions of maxillary anterior teeth and increased overjet.^9^ ^,^ ^10^ Other pathological signs such as dental caries and discolorations and xerostomia are also frequently reported.^11^ ^,^ ^12^

In order to deal with various dental conditions, a conservative treatment approach is preferred; while for the correction of various orthodontic dentofacial deformities and malocclusion, specialized interventions - such as the use of different orthodontic appliances (fixed , removable, functional, extraoral appliances) and, in advanced cases, surgical approach- is required.^13,14^ Considered a “global health burden” this issue has been neglected due to its expensive and long term treatment requirements.^15^ Furthermore, there are no comprehensive guidelines given by the Thalassemia International Foundation for management of oral health complications in such patients.^16^

Therefore, it is of paramount importance to assess the prevalence and orthodontic treatment needs in this particular lot of population, so that a generalized opinion may be formulated regarding the severity of malocclusion, burden of disease and required expertise and resources. Hence, the primary objective of this study was to evaluate the prevalence of different malocclusion characteristics in the children suffering from β -thalassemia and to assess the orthodontic treatment needs using Grainger’s Treatment Priority Index (TPI)^17^ and index of orthodontic treatment need (IOTN) - dental health component (DHC).^18^

## MATERIAL AND METHODS

A cross-sectional study was conducted at the department of orthodontics, Bakhtawar Amin Medical and Dental College in collaboration with Sundas Foundation. Ethical approval was obtained from the Institutional Research Board, Bakhtawar Amin Dental College and Hospital, Multan (protocol #309/2020), prior to the data collection. 

### Sample selection

A total of two-hundred transfusion-dependent children suffering from β-thalassemia major registered with Blood Bank and Hematological Services Charitable Organization were included in the study. A non-probability consecutive sampling technique was employed.

The inclusion criteria comprised of subjects diagnosed with homozygous β-thalassemia, within the age range of 11 to 17 years, receiving regular blood transfusions. Children in mixed dentition period, or those having history of any previous orthodontic treatment or any type of chronic disease or growth retardation were excluded from the study. 

For the control group, a sample of 200 healthy school children, matched according to age and gender, were selected from four different schools, using stratified sampling technique, and the TPI and IOTN-DHC data was recorded. The same exclusion criteria were followed for the controls as was for the study group.

### Data collection

For the evaluation of TPI and IOTN-DHC, the main investigator was trained and the intraoral examination of all the patients, in cases and control categories, was performed by the same examiner. The data collection form recorded the patient’s demographic details, IOTN-DHC grade and various parameters of TPI. The assessment of TPI parameters was carried out as follows:

» Bilateral first molar relationship depicts sagittal relation between the maxillary and mandibular first molars, and is considered normal if the mesiobuccal cusp of maxillary first molar occludes in the buccal groove of mandibular first molar. First molar relationship is defined by a constant in TPI that reflects the severity of malocclusion.
» Overjet was measured as the horizontal distance from the labial surface of the most prominent maxillary central incisor to the labial surface of mandibular central incisor, parallel to the occlusal plane.
» Overbite was measured as the amount of vertical overlap on the mandibular central incisor by the maxillary central incisor, considering the extent of coverage of crown height of mandibular central incisor. A negative overbite depicted no overlap between maxillary and mandibular incisors, and was denoted as open bite. Open bite was measured as the vertical distance between maxillary and mandibular incisors.
» Tooth displacement: was assessed as the number of teeth noticeably rotated or displaced from ideal alignment, and posterior teeth rotated above 45° or anterior teeth displaced greater than 2 mm were given double weightage.
» Crossbite is a buccal or lingual displacement of the posterior teeth, deviating from normal cusp-fossa relationship. During TPI assessment, the number of teeth in buccal or lingual crossbite was assessed and were given respective weightages, as shown in Table 1.

**Table 1:** Treatment Priority Index^17^.

			(6) Distocclusion					(7) Mesiocclusion					
First Molar Relationship (choose the appropriate column)			2 sides full Class II	1 side half Class II 1 side full Class II	2 sides half Class II or 1 side full Class II	1 side half Class II	N E U T R A L	1 side half Class III	2 sides half Class III or 1 side full Class III	1 side half Class III 1 side full Class III	2 sides full Class III	W E I G H T	Type of Syndrome
Incisor Horizontal Relationship in mm													
(1) Upper Overjet		>9	2.0	3.4	5.4	9.3	10+	9.3	5.4	3.4	2.0		Retrognathism
		9	1.4	2.5	4.0	6.9	10+	6.9	4.0	2.5	1.4		
		8	1.0	1.8	2.8	4.8	8.0	4.8	2.8	1.8	1.0		
		7	0.6	1.1	1.8	3.0	5.1	3.0	1.8	1.1	0.6		
		6	0.4	0.6	1.0	1.7	2.9	1.7	1.0	0.6	0.4		
		5	0.2	0.3	0.4	0.8	1.3	0.8	0.4	0.3	0.2		
NORMAL (Counting 0)													
(2) Lower Overjet		1	0.2	0.3	0.4	0.8	1.3	0.8	0.4	0.3	0.2		Prognathism
		0	0.4	0.6	1.0	1.7	2.9	1.7	1.0	0.6	0.4		
		1	0.6	1.1	1.8	3.0	5.1	3.0	1.8	1.1	0.6		
		2	1.0	1.8	2.8	4.8	8.0	4.8	2.8	1.8	1.0		
		3	1.4	2.5	4.0	6.9	10+	6.9	4.0	2.5	1.4		
		>3	2.0	3.4	5.4	9.3	10+	9.3	5.4	3.4	2.0		
Incisor Vertical Relationship													
(3) Overbite in relation to crown thirds		>3/3	2.9	3.8	4.8	6.2	8.0	6.2	4.8	3.8	2.9		Overbite
		3/3 to ⅔	1.5	2.0	2.4	3.2	4.1	3.2	2.4	2.0	1.5		
		⅔ to ⅓	0.5	0.7	0.9	1.1	1.5	1.1	0.9	0.7	0.5		
NORMAL (Counting 0)													
(4) Open bite in mm		<2	1.5	2.0	2.4	3.2	4.1	3.2	2.4	2.0	1.5		Open bite
		2 to 4	2.9	3.8	4.8	6.2	8.0	6.2	4.8	3.8	2.9		
		>4	4.9	6.3	7.9	10+	10+	10+	7.9	6.3	4.9		
(10) Teeth displacement		No.											
• Sum of teeth rotated 45^o^ or 2mm displaced • Sum of teeth rotated >45^o^ or >2mm displaced x2 • Total (0,1 without counting)		2	0.1	0.1	0.2	0.3	0.4	0.3	0.2	0.1	0.1		Distocclusion and/or posterior buccal crossbite May be: YES: - maxilla - expansion - Brodie syndrome NO: - maxilla - collapse - posterior crossbite
		3	0.2	0.3	0.4	0.7	1.1	0.7	0.4	0.3	0.2		
		4	0.3	0.5	0.9	1.2	1.9	1.2	0.9	0.5	0.3		
		5	0.5	0.8	1.2	1.9	3.0	1.9	1.2	0.8	0.5		
		6	0.7	1.1	1.8	2.8	4.3	2.8	1.8	1.1	0.7		
		7	1.0	1.5	2.4	3.9	5.9	3.9	2.4	1.5	1.0		
		8	1.3	19	3.1	4.9	7.7	4.9	3.1	19	1.3		
		9	1.7	2.5	4.1	6.2	9.7	6.2	4.1	2.5	1.7		
		>9	2.0	3.0	4.9	7.7	10+	7.7	4.9	3.0	2.0		
Constants			5.17		3.95	2.72	1.50	0.27	1.50	2.72	3.95	5.17	
(8) Sum of teeth in posterior crossbite	Buccal upper posterior teeth	No.	0	1	2	3	4	5	6	7	8	More	
		Weight	0	0.1	0.6	1.3	2.2	3.5	5.0	6.9	9.0	10	
	Lingual upper posterior teeth	No.	0	1	2	3	4	5	6	More			
		Weight	0	0.3	1.0	2.3	4.2	6.5	9.4	10			
Score sum is the Treatment Priority Index (TPI)													

In addition to these parameters, patients were examined for congenitally missing incisors and other intraoral defects. The findings of TPI were recorded and a total TPI score was computed for each subject using the Table 1. The total TPI score for each subject was calculated and graded according to the Malocclusion Severity Estimate (MSE), as given below.

- Virtually classic normal occlusion: TPI score <1.
- Minor manifestations of malocclusion and treatment need is slight: TPI score from 1 to 3.99
- Definite malocclusion, but treatment elective: TPI score from 4 to 6.99
- Severe handicap, treatment highly desirable: TPI score from 7 to 9.99
- Very severe handicap with treatment mandatory: TPI score >10.

### Reliability of measurements

The TPI scores and IOTN-DHC for the 30 patients were re-graded after one month to assess intraexaminer reliability. Intraclass correlation coefficients were performed for TPI, which showed a correlation coefficient of 0.89. The Kappa statistics were applied for IOTN-DHC, which showed a high coefficient of reliability (0.957). Thus, both assessments were found to have good intraexaminer reliability. 

### Statistical analysis

Independent sample *t*-test was used to compare the linear variables -like TPI scores, overjet and overbite- between the cases and controls. Chi-square test was used to compare the frequency of IOTN-DHC grades, Angle’s classification and other categorical variables, between cases and controls. A value of *p*<0.05 was taken as statistically significant. 

## RESULTS

A total of 121 male and 79 female adolescents were included in the study group, while 107 males and 93 females were included in the control group. There was no statistically significant difference (*p*=0.189) in the gender distribution between the two groups. The mean ages of children in the study and control groups were 13.89 ± 1.79 and 14.1 ± 2.07 years, respectively. There was no statistically significant difference (*p*=0.258) in the mean ages of the children belonging to both the groups. 

The frequency of children with Angle’s Class I malocclusion in thalassemic children was far less than that in normal children (38% *vs.* 67.5%). On the other hand, the prevalence of Angle’s Class II malocclusion was 59% in thalassemic children, as compared to 24.5% in normal children. Table 2 compares the prevalence of Angle’s malocclusion between the cases and controls. 

**Table 2:** Comparison of Angle’s classification between cases and controls.

Angle’s classification of molar relationship	Thalassemic children (n=200)	Healthy controls (n=200)	Total	p value
Class I	76	135	173	<0.001
Class II	118	49	108	
Class III	6	16	19	

**Figure 1:**
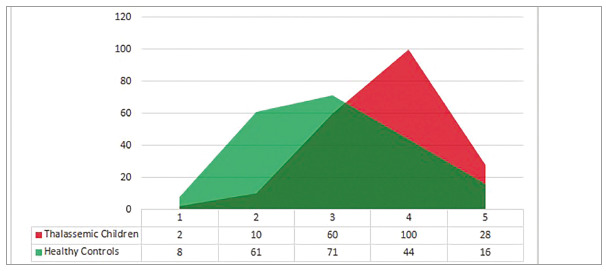
Distribution of cases and controls into five grades of IOTN-DHC.

A greater proportion of thalassemic children were in IOTN grades 3 and 4, as compared to controls (*p*<0.001). These results were highly significant. The distribution of cases and controls in different IOTN grades is given in Figure 1.

There were significant differences (*p*<0.001) in the mean overjet and overbite between the cases and controls (Table 3). Moreover, the prevalence of mild tooth displacements (rotation up to 45 degree or displacement up to 2mm) and severe tooth displacements (rotation >45 degree or displacement >2mm) were 1.8 times and 3.5 times greater in the cases, as compared to the controls, respectively. 

**Table 3:** Comparison of different occlusal parameters between cases and the controls.

	Thalassemic children n=200	Healthy controls n=200	Mean difference	p value
Overjet (mm)	4.17 ± 2.64	2.90 ± 2.31	1.27	<0.001*
Overbite (mm)	4.18 ± 2.88	3.14 ± 1.91	1.04	<0.001*
Teeth mildly displaced	4.37 ± 3.10	2.49 ± 2.05	1.88	<0.001**
Teeth severely displaced	2.09 ± 2.64	0.59 ± 0.95	1.50	<0.001**
Number of patients with buccal/lingual crossbite	20	8	6%	0.079**

* Independent sample t-test. ** Chi-square test.

**Figure 2:**
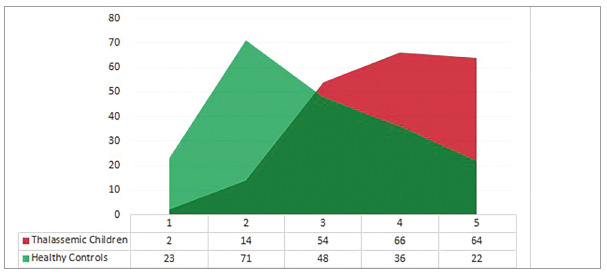
Distribution of cases and controls into TPI grades of MSE.

According to TPI, majority of thalassemic children had MSE grade 4 and 5 malocclusion, as compared to grade 2 and 3 in controls. Severe forms of malocclusions were significantly (*p*<0.001) more prevalent in thalassemic children, as compared to the controls (Fig. 2). The mean TPI score for thalassemic children was 8.55 ± 4.13, which was significantly higher (*p*<0.001) than that in normal children, which was recorded as 4.09 ± 3.27.

## DISCUSSION

Patients suffering from β-thalassemia major usually become dependent on regular blood transfusions early in life. However, the dental features become noticeable when the permanent teeth start to erupt and child enters puberty. So, patients aged 11-17 years were included in this study, which also marks the time when orthodontic treatment is usually sought. 

There are several indices to evaluate the severity of malocclusion, such as American Board of Orthodontics Discrepancy Index (ABO-DI), Summer’s Occlusal Index, and Grainger’s TPI. ABO-DI requires cephalometric assessment along with the clinical examination, which makes it impractical to apply for patient when no actual treatment is being provided. On the other hand, occlusal index is relatively complicated and requires more clinical time, as compared to TPI.^19^ Thus, we used Grainger’s TPI, which is not only reproducible and valid, but has also proven to be a useful tool for epidemiological assessment of malocclusion.^20^ ^,^ ^21^

**Figure 3:**
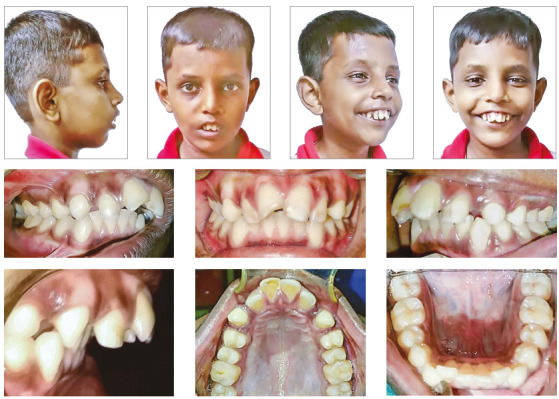
Patient presenting convex facial profile with incompetent lips. Note fractured edge of maxillary right central incisor, which is not an uncommon finding in patients with excessive proclination of maxillary incisors.

**Figure 4:**
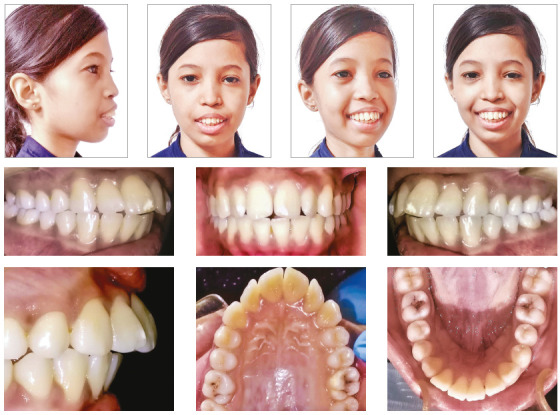
A typical female patient showing features of skeletal Class II malocclusion due to maxillary prognathism, Class II division 1 malocclusion with proclined incisors and an increased overjet.

In the present study, the subjects with thalassemia were found to have greater prevalence of skeletal Class II, increased overjet and both mild and severe tooth displacements, as compared to the controls. In the extraoral aspect, these children had a tendency towards convex facial profile due to maxillary prognathism and incompetent lips. The typical extraoral and intraoral clinical features of transfusion-dependent children with β-thalassemia are shown in Figures 3 and 4. Enlarged maxillary bone marrow due to extracellular haematopoiesis and lack of oral hygiene awareness may well have contributed to the aforementioned malocclusion. The statistics from the current and previous studies reveal that thalassemic patients consistently have greater prevalence of Angle’s Class II malocclusion, reported to be 55% by Gupta et al,^22^ and 51% by Shahsevari et al,^23^ as compared to 59% in the current study. 

The statistics also reveal that around 84% and 65% of the thalassemic patients lie in the IOTN moderate to severe treatment need groups, and MSE handicapping and severely handicapping malocclusion groups, respectively. Thus about two-thirds to three-fourths of transfusion-dependent thalassemic children required definitive orthodontic treatment. Gupta et al^22^ used Grainger’s TPI and found that 68% of their thalassemic sample had definitive to severely handicapping malocclusion. The current study reports that 29% of healthy children had handicapping to severely handicapping malocclusion. The reported percentage of healthy school children needing definitive orthodontic treatment ranges from 18 to 26 percent in other studies, which is similar to the present findings.^22^ ^,^ ^24^ ^,^ ^25^

The similarities between the present results and those of Gupta et al^22^ are remarkable. This might be due to similar selection criteria and subcontinental origin of the sample. Majority of the thalassemic patients in the world are located in South Asian countries like India, Pakistan and Bangladesh.^26^ According to one estimate, about 10 million individuals in Pakistan suffer from thalassemia minor, while more than 50,000 have thalassemia major.^27^ ^,^ ^28^ Most of these patients are being treated by nongovernmental organizations, which have limited resources. The sample of thalassemic children in the current study was also collected from transfusion centers run by one of such organizations. A single center study reports that the treatment provided at these centers is not optimum, and majority of the thalassemic children are under-transfused.^29^ This might explain greater severity of malocclusion in the present sample, as compared to that reported in literature for other population groups.^22^ ^,^ ^23^

As the dental and orthodontic complications are not life threatening, they are likely to be ignored by the affected individual and the medical specialists. As a consequence, these problems tend to worsen over time. A preventive and interceptive orthodontic treatment approach is required in these patients, to reduce the likelihood of trauma, to improve stomatognathic function and the facial appearance.^30^ In the present sample, the age at first transfusion ranged from 6 months to 6 years. Hakeem et al^31^ have shown that starting transfusions in older age is a protective factor against poor quality of life. Authors recommend that the relationship between the severity of malocclusion in these children and the age of starting transfusions should be assessed on a larger sample. 

Effective orthodontic treatment interventions usually comprise of high-pull headgear and functional appliances, to restrict maxillary sagittal and vertical growth, as well as to enhance mandibular growth. As these patients usually have to undergo regular blood transfusions, hence orthognathic surgeries need to be avoided if possible.^32^ Thus, early intervention, interceptive treatment and growth modifications are usually the treatment of choice in these patients.^30^ ^,^ ^32^

The dental and orthodontic treatment modalities may significantly contribute to the generalized well-being and improved quality of life in these patients. Based on the staggering findings of the current study, it is recommended to have continuous orthodontic supervision for transfusion-dependent thalassemic children, similar to those for the patients with craniofacial syndrome and cleft lip and palate. 

## CONCLUSION

Majority of thalassemic children suffer from Class II malocclusion; in contrast to normal children, who most commonly have Class I malocclusion. The IOTN-DHC grades and TPI-MSE depict that the malocclusion in thalassemic children is much more severe and handicapping. Since the life expectancy of these children has improved over the last few decades, more expertise and resources need to be allocated to cater the orthodontic treatment needs of these children.
